# Studying moderators of implementation: analysis from an intervention to reduce disrespect and abuse in facility-based childbirth

**DOI:** 10.1186/1472-6963-14-S2-P100

**Published:** 2014-07-07

**Authors:** Kate Ramsey, Wema Moyo, Selemani Mbuyita, Stephanie Kujawski, Godfrey Mbaruku, Lynn P Freedman

**Affiliations:** 1 AMDD, Mailman School of Public Health at Columbia University, New York, NY, USA; 2 Ifakara Health Institute, Dar es Salaam, Tanzania

## Background

Across the globe, women who deliver in health facilities report experiencing disrespect and abuse (D&A). In low- and middle-income countries, D&A is particularly catastrophic because it may cause women to opt against facility delivery, as well as violate their human rights. D&A is likely a frontline manifestation of multi-level problems in complex health systems; yet efforts to address D&A have typically focused on micro-levels - either health providers’ ethics or users’ demand for quality care. These have rarely achieved sustainable implementation [1], mirroring clinical quality improvement challenges. Implementation science holds promise for investigating these challenges. The Consolidated Framework for Implementation Research (CFIR) assembles constructs from across the literature that can guide inquiry [2].

## Materials and methods

The Staha Project studies the magnitude and dimensions of D&A, and is testing mechanisms for its mitigation. It is based in two Tanzanian districts, with one assigned to intervention. Implementation is conducted by four facilities, catchment communities and local leadership. Implementation research includes patient and provider satisfaction surveys, observations, reports and qualitative interviews. Relevant CFIR constructs were selected to develop the lines of inquiry and as themes for qualitative analysis adapted iteratively based on data. We conducted descriptive analyses of quantitative data.

## Results

The intervention was developed through a participatory process grounded in baseline research to address meso- and micro-level drivers at the district level. A change process was elaborated including activation of a client service charter and a facility-based change process. Mutuality of respect emerged as the underlying value for the process. Results from the planning process and the first year of implementation will be presented using CFIR constructs. These will include findings related to the characteristics of the intervention, inner and outer settings, individual implementers and the process.

## Conclusions

The CFIR was a useful tool to establish lines of inquiry and frame analysis. Ongoing analysis permitted identification of areas for improvement. We found the strongest constructs were regarding the intervention, the individual, and the inner setting characteristics. The outer setting construct could be further developed, especially for interventions that go beyond health facilities.

## References

[B1] Bowser D Hill K Exploring Evidence for Disrespect and Abuse in Facility-Based Childbirth: Report of a Landscape Analysis 2010 Washington, DC: USAID-TRAction Project

[B2] Damschroder LJ Aron DC Keith RE Kirsh SR Alexander JA Lowery JC Fostering implementation of health services research findings into practice: a consolidated framework for advancing implementation science Implement Sci 2009 14 50 10.1186/1748-5908-4-50 19664226 PMC2736161

